# Meta-analysis cum machine learning approaches address the structure and biogeochemical potential of marine copepod associated bacteriobiomes

**DOI:** 10.1038/s41598-021-82482-z

**Published:** 2021-02-08

**Authors:** Balamurugan Sadaiappan, Chinnamani PrasannaKumar, V. Uthara Nambiar, Mahendran Subramanian, Manguesh U. Gauns

**Affiliations:** 1 Plankton Ecology Lab, Biological Oceanography Division, CSIR-National Institute of Oceanography, Dona Paula, Panaji, Goa, 403004 India; 2 Department of Bioengineering, Imperial College London, South Kensington, London, SW72AZ UK; 3 Department of Computing, Imperial College London, South Kensington, London, SW72AZ UK; 4 Faraday-Fleming Laboratory, London, W148TL UK

**Keywords:** Data processing, Machine learning, Microbial ecology, Microbial communities, Metagenomics, Microbiome

## Abstract

Copepods are the dominant members of the zooplankton community and the most abundant form of life. It is imperative to obtain insights into the copepod-associated bacteriobiomes (CAB) in order to identify specific bacterial taxa associated within a copepod, and to understand how they vary between different copepods. Analysing the potential genes within the CAB may reveal their intrinsic role in biogeochemical cycles. For this, machine-learning models and PICRUSt2 analysis were deployed to analyse 16S rDNA gene sequences (approximately 16 million reads) of CAB belonging to five different copepod genera viz., *Acartia* spp., *Calanus* spp., *Centropages* sp., *Pleuromamma* spp., and *Temora* spp.. Overall, we predict 50 sub-OTUs (s-OTUs) (gradient boosting classifiers) to be important in five copepod genera. Among these, 15 s-OTUs were predicted to be important in *Calanus* spp. and 20 s-OTUs as important in *Pleuromamma* spp.. Four bacterial s-OTUs *Acinetobacter johnsonii*, *Phaeobacter, Vibrio shilonii* and Piscirickettsiaceae were identified as important s-OTUs in *Calanus* spp., and the s-OTUs *Marinobacter, Alteromonas, Desulfovibrio, Limnobacter, Sphingomonas, Methyloversatilis, Enhydrobacter* and Coriobacteriaceae were predicted as important s-OTUs in *Pleuromamma* spp., for the first time. Our meta-analysis revealed that the CAB of *Pleuromamma* spp. had a high proportion of potential genes responsible for methanogenesis and nitrogen fixation, whereas the CAB of *Temora* spp. had a high proportion of potential genes involved in assimilatory sulphate reduction, and cyanocobalamin synthesis. The CAB of *Pleuromamma* spp. and *Temora* spp. have potential genes accountable for iron transport.

## Introduction

Copepods (Subphylum Crustacea; Class Hexanauplia; Subclass Copepoda) are an abundant and diverse group of zooplankton in the ocean^1,2^. They play a key role in energy transfer within the pelagic food web^3^. They are also well-known for their wide-ranging and flexible feeding approaches^4^. Copepods, usually not more than a few millimetres in length, support a wide range of bacterial communities, both internally and externally (due to the release of organic and inorganic nutrients during feeding and excretion)^1–3^. In addition, it is an already-established fact that there is an exchange of bacterial communities between the copepods and the water-column, due to their feeding behaviour^5,6^, and copepods transfer microbes from the photic zone up to the middle of the twilight zone^3,7,8^. The different environmental conditions between the surrounding water and copepods favour different bacterial communities^6,7,9^.

However, feeding also changes the composition of bacterial communities in the copepod gut, *e.g.*, a high abundance of Rhodobacteraceae was reported in *Acartia* sp. with a full gut, in comparison with its starved counterparts^10^. Copepods have mutualistic associations with (Gammaproteobacteria) *Pseudoalteromonas* spp.. In addition, Gammaproteobacteria was found to be more abundant in starved *Centropages* sp., *Acartia* sp.^10^ and *Pleuromamma* sp.^11^. A notable change was observed among bacterial communities between the diapause phase and actively-feeding *Calanus finmarchicus*^2^. Similarly, Flavobacteriaceae was meagre in copepods during diapause and abundant in its actively-feeding counterparts^2^. Datta et al.^2^ reported that *Marinimicrobium* (Alteromonadaceae) was relatively more abundant in deep-dwelling copepods than in its shallow counterparts, and concluded that the copepods have inter-individual microbiome variations; however, the factors driving these variations are still unknown. From these early reports, it is well-known that bacterial communities associated with copepods vary according to many factors, based on feeding, difference in stages of life, body size, and their vertical migration through the water column. Moreover, there may be a particular relationship or symbiosis, and a natural core microbiome that depends not necessarily on the food, but on the host environment^10^. Herein, the term 'bacteriobiome' means the total bacterial composition inhabiting a specific biological niche (for example, copepods), including their genomic content and metabolic products^12^. It is a well-known fact that host-associated microbial communities remain essential for maintaining any ecosystem, and any variation in these communities may be unfavourable. Thus, studying the specific bacterial taxa associated with copepods and its variations, as well as analysing potential genes within the copepod-associated bacteriobiomes (CAB), will help us in understanding their role in the host’s health, marine food web and biogeochemical cycles.

Until now, only a few studies have sought to identify the core-bacteria associated with the copepods, using their clustering patterns^2^ and presence/absence data^1^. From these studies, approximately eight bacterial orders, such as Actinomycetales, Bacillales, Flavobacteriales, Lactobacillales, Pseudomonadales, Rhizobiales and Vibrionales, were identified as core members in *Pleuromamma* spp.^1^, whereas the phylum Proteobacteria were identified as core operational taxonomic units (OTUs; equivalent to species), along with Actinobacteria and Bacteroidetes in *Calanus finmarchicus*^2^.

Moreover, the gut of copepods has an acidic pH and a different oxygen gradient from the anal opening to the metasome region; this may influence certain groups of bacteria to colonise within the copepods. These bacterial communities could be specialised in iron dissolution, anaerobic methanogenesis^13^, nitrite reduction^14^ and anaerobic dinitrogen (N_2_) fixation^15^. At any given time, the abundance of CAB will be an order of two to three less than seawater, but, if we assume that there is one copepod per litre of seawater, the contribution of CAB to marine biogeochemical cycles will be significant^1^. Already, various studies have shown that CAB has a potential role in biogeochemical processes such as nitrogen fixation^15,16^, denitrification^9^, sulphur^17^ and iron mineralisation^13^.

The masking effect of the abundant bacterial community, associated with copepod diet, copepod life stage and environmental conditions, was considered to be the main hindrance in defining core bacterial OTUs specific to copepod genera^2,10^. Herein, we combined the data from previous studies that dealt with CAB, and used machine learning algorithms to understand the core bacteria associated with the copepods at least up to the genus level. For this, we analysed 16S rDNA gene sequences (V3–V4 & V4–V5 regions; ~ 16 million reads) of CAB belonging to five different copepod genera (*Acartia* spp., *Calanus* spp., *Centropages* sp., *Pleuromamma* spp. and *Temora* spp.) using the Quantitative Insights into Microbial Ecology (QIIME2) package^18^. In addition, we hypothesised that, if the copepod genera have specific OTUs, then different copepods will have a distinctive CAB, and the biogeochemical potential of the CAB will differ. We used random forest classifier, gradient boosting classifier, principal coordinate analysis (PCoA), analysis of the composition of microbiome (ANCOM), principal component analysis (PCA) and phylogenetic investigation of communities by reconstruction of unobserved states (PICRUSt2) analysis^19^ to test this hypothesis. The present study represents one of the biggest CAB-related DNA sequence data analysed to date.

## Materials and methods

### Data collection

We systematically reviewed the studies related to CAB. The relevant published research articles were searched and retrieved from PubMed, Google Scholar and SCOPUS, using keywords such as copepods gut microbiome, copepod associated bacteria/microbiome, copepods gut flora, copepod microbiome and zooplankton associated microbiome on Jan 30th, 2020. Aside from the search for published research articles, we also searched in public databases (for published Ion Torrent, Pyro, and Illumina sequence data), such as the NCBI-SRA, ENA, DDBJ-DRA and Figshare, using the above-mentioned keywords.

Overall, 11 study data were retrieved for meta-analysis (Table 1) containing 514 next-generation sequence libraries. We pre-processed separately every individual file within the study and prepared a quality control (QC) report.

**Table 1:** List of sequence libraries representing the copepod-associated bacteriobiomes (CAB). Of these, only seven libraries (highlighted in bold) were analysed in this study.

S. no.	NCBI BioProject no.	Species name	16S rDNA region	Sequencing platform	Reference
1	PRJNA383099	Details not available	Details not available	Illumina MiSeq	No
2	**PRJEB23400**	*Pleuromamma* sp.	V3–V4	Illumina	No
3	**PRJNA416766**	*Acartica* sp. and *Temora* sp.	V3–V4 & V4–V5 (archaea)	Illumina MiSeq	Wage et al.^37^
4	**PRJNA341063**	*Pleuromamma* spp.	V3–V4	Illumina MiSeq	Shoemaker and Moisander^1^
5	**PRJNA285993**	*Acartia longiremis,Centropages hamatus,* and *Calanus finmarchicus*	V3–V4	Illumina MiSeq	Moisander et al.^10^
6	PRJEB8785	*Acartia tonsa* and *Centropages hamatus*	V3–V4	454/FLX-based	Skovgaard et al.^69^
7	PRJNA248671	*Undinula vulgaris, Pleuromamma* spp., *Sapphirina metalina*, *Pseudocalanus* spp. and *Tigriopus* sp.	V5–V9	454 GS FLX Titanium	Shoemaker and Moisander^70^
8	PRJEB14826	*Acartia tonsa* and *Temora longicornis*	V3–V4	Illumina MiSeq	Dorosz et al.^47^
9	**PRJNA322089**	*C. fimaarchincus*	V4	Illumina MiSeq	Datta et al.^2^
10	**PRJDB5552**	*Calanus* sp., *Paraeuchaeta* sp., *Themisto* sp., *Evadne* sp., and *Oncaea* sp.	V3–V4	Illumina MiSeq	De Corteet al.^9^
11	PRJNA433804	*Spaniomolgus* sp.	V4–V5	Ion_Torrent	Shelyakin et al.^71^

### Pre-processing

The sequence quality was checked using the FastQC tool^18^, and the minimum base per quality for future analysis was fixed as PHRED > 25. Based on the QC, high rates of erroneous sequences from Illumina, 454 and Ion Torrent files (Table 1) were removed from the meta-analysis. The two major reasons for exclusion were (1) erroneous sequences (of PHRED < 25) and (2) short reads (< 200 bps) screened by DADA2^20^ while picking sub-OTUs (s-OTUs). Overall, Illumina sequences were of quality than the Ion-torrent and Pyrosequence sequences. Finally, we carried out a meta-analysis with 452 files of CAB in order to test the proposed hypothesis.

### Meta-analysis

#### Sequence screening and preparations for meta-analysis

We used QIIME2 version 2019.10^18^ for the meta-analysis. QIIME2 pipeline provides a start-to-finish workflow, beginning with demultiplexing sequence reads and finishing with taxonomic and phylogenetic profiles. The sequences from the individual study were imported to QIIME2 using CasavaOneEight format, and the quality of the sequences was checked using the default settings in QIIME2. Based on the sequence quality, the sequence was trimmed, denoised, aligned and checked for chimera using DADA2 (single and paired-ends sequences were trimmed based on the length of primer used)^20^. The feature table and representative sequence of each file were merged using the QIIME2 feature merge table, and representative sequences were merged.

#### Taxonomic classification

The merged files were aligned to phylogeny against the Greengenes reference sequence sepp-refs-gg-13-8 using q2-fragment-insertion^21^. Incorrect taxonomic and phylogenetic assignments, due to differences in 16S rDNA hypervariable regions and to merging of variable lengths during analysis, were solved using q2-fragment insertion technique (SATe-enabled phylogenetic placement in QIIME2 plugin)^21^. The core diversity was calculated before (to calculate the impact on diversity) and after removing mitochondria (mtDNA) and chloroplast (clDNA) sequences from the datasets. The mtDNA- and clDNA-filtered datasets were used for calculating diversity, taxonomy, important (core) s-OTUs, and the difference in composition estimation using QIIME2 and the diversity graph was plotted within QIIME2. We used Unweighted, Weighted UniFrac and Jaccard distance matrices to compute the beta diversity, and the outcomes were envisaged using PCoA in QIIME2. A permutational multivariate analysis of variance (PERMANOVA)^22^ through the Unweighted, Weighted UniFrac, along with Jaccord distance-based beta-diversity, was calculated within QIIME2. We used a standard pre-trained Greengenes reference dataset (gg_13_8_99_OTU_full-length)^23^, SILVA reference database (SILVA_188_99_OTUs full-length)^24^ and a fragment-insertion reference dataset (ref-gg-99-taxonomy). We then decided to discuss the results from the fragment-insertion reference dataset.

We also implemented ANCOM^25^ in QIIME2 plugin to identify the significantly different bacteria between the copepod genera. ANCOM used F-statistics and W-statistics to determine differences, where W represents the vigour of the ANCOM test for the tested number of species and F represents the measure of the effect size difference for a particular species between the groups (copepods). In order to predict the important bacteria associated with the copepods, we used a sophisticated supervised machine learning classifier (SML): RandomForest Classifier (RFC)^26^ and Gradient Boosting Classifier (GBC)^27^ using built-in QIIME2. RFC is one of the most accurate for managing large and noisy datasets. This learning algorithm often manages unbalanced sample distributions, and is less susceptible to overfitting and generating unbiased classifiers^28^. The gradient boosting method involves the use of several weak learners by taking the loss function from the previous tree and using it to enhance the classification. This technique is less prone to overfitting and does not suffer from the dimensionality curse, but is susceptible to noisy data and outliers^29^.

The mtDNA and clDNA filtered feature table and representative sequences were also used as an input for predicting CAB potential metabolic function using PICRUSt2^19^. The output abundance KEGG data were analysed in statistical analysis of metagenomic profile (STAMP), which includes PCA^30^, to find the significant difference in potential functions of CAB between the copepod genera using the Kruskal–Wallis H-test^31^ with Tukey–Kramer parameter^32^. The KEGG metabolic maps^33–35^ were used as a reference from which to draw the figure representing the copepod genera with a high proportion of potential functional genes.

### Copepod phylogeny

The 18S rDNA gene sequences of five copepod genera (used in the present study) were extracted from the Genbank (NCBI). These sequences were aligned and the consensus representative sequence from each genus was obtained using Mega X version 10.1.7. These consensus sequences were used for studying the phylogenetic relationship between the copepods at genera level, using neighbour-joining tree in Mega X^36^.

## Results

The present study represents one of the largest CAB-related DNA sequence data analyses to date. New bioinformatics tools have been created to cope with data generated by the next-generation sequencers. To overcome the bias in the tools, we used standard, well-recognised pipelines, such as FastQC and QIIME2 demultiplexing statistics, for reading the quality of each sequence, and the DADA2 algorithm for clustering, aligning, and filtering of chimeric sequences^20^. From the collected data, 12% (n = 62, i.e., 35 Roche, six Ion Torrent and 21 Illumina-generated sequence files, Table 1) of the files failed during the QC and were omitted. Ultimately, 452 raw files belonging to five different copepod genera were subjected to analysis.

### DNA sequence data analysis

From the 452 raw files, we analysed ~ 16 million V3–V4 regions, (except 18 files of V4-V5 archaea specific primer files from Wage et al.^37^, Table 1) of bacterial 16S rDNA gene sequences belonging to five copepod genera: *Acartia* spp., *Calanus* spp., *Centropages* sp., *Pleuromamma* spp. and *Temora* spp.. After quality filtering through the DADA2 package, between 0.29 and 8.23% of sequences were removed (Table 2), and a total of 12,139,998 non-chimeric sequences were used for downstream analysis.

**Table 2:** Details of the number of Illumina files, sequences extracted, quality filtered (Phred score < 25) and non-chimeric sequences. RP indicates 'relative proportion'.

Species	No. of files	RP of files (%)	Gross Sequences	RP of gross sequences (%)	Net. no. of sequences after DADA2 denoise	RP of seqs after DADA2 denoise (%)	Net. no. of non-chimeric seqs	RP of non-chimeric seqs (%)	Net. no. of seqs lost after DADA2 chimera filter	RP of seqs loss after DADA2 chimera filter (%)
*Acartia* spp.	30	6.63	2,583,086	16.18	2,278,085	14.27	2,013,811	12.61	569,275	3.56
*Calanus* spp.	246	54.42	6,658,845	41.71	5,837,666	36.56	5,418,497	33.94	1,240,348	7.76
*Pleuromamma* spp.	148	32.74	4,310,670	27.00	3,204,613	20.07	2,995,684	18.76	1,314,986	8.23
*Centropages* sp.	12	2.65	798,974	5.00	783,422	4.90	752,340	4.71	46,634	0.29
*Temora* spp.	16	3.53	1,612,300	10.09	1,340,853	8.39	959,666	6.01	652,634	4.08
Total	452		15,963,875		13,444,639		12,139,998		3,823,877	

### CAB alpha diversity

**Figure 1:**
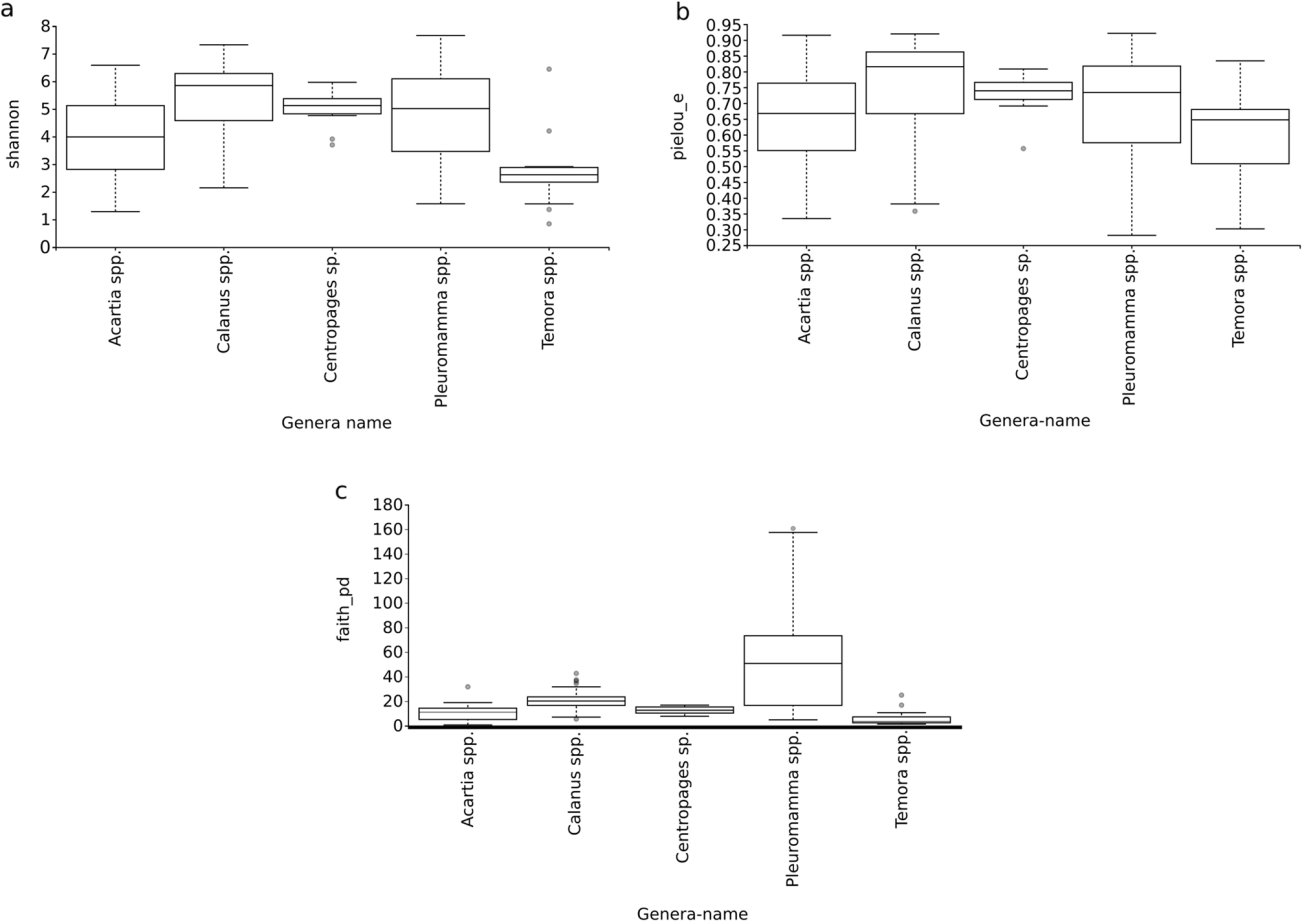
Alpha diversity composition and variation, (**a**) Shannon index (Richness and diversity accounting for both abundance and evenness of the taxa present); (**b**) Evenness index (Relative evenness of species richness); (**c**) Faith’s Phylogenetic Diversity index (biodiversity incorporating phylogenetic difference between species) corresponding to the CAB within five different copepod genera.

From the bacterial diversity Shannon ('H') indices for the five copepod genera, *Calanus* spp. showed the maximum (median, Q1–Q3: 5.85, 4.58–6.29) abundance and evenness of CAB, followed by *Centropages* sp. (5.13, 4.81–5.41) and the least was observed in *Temora* spp. (2.62, 2.36–2.89) (Fig. 1a).

The Kruskal–Wallis analysis revealed that the H index of the CAB within the *Acartia* spp. was significantly different from that of *Calanus* spp., *Centropages* sp., *Temora* spp. and *Pleuromamma* spp., with a p-value ranging between 0.000002 and 0.023779 (Fig. 1a). The H index of the CAB within the *Temora* spp. was significantly different from that of *Centropages* sp. (p = 0.0012) and *Pleuromamma* spp. (p = 0.000209). The H index of the CAB within the *Calanus* spp. was significantly different from that of *Centropages* sp., *Pleuromamma* spp. and *Temora* spp., with a p-value ranging between 0.000008 and 0.05.

Evenness indices showed that CAB of the *Calanus* spp. (0.82, 0.67–0.86) have a high evenness index, followed by *Centropages* sp. (0.74, 0.71–0.77), *Pleuromamma* spp. (0.73, 0.57–0.82), and least in *Temora* spp. (0.65, 0.51–0.68) (Fig. 1b).

The Kruskal–Wallis analysis of CAB evenness index was calculated for all copepod genera (pairwise). There was a significant different evenness (p-value ≤ 0.05) between the CAB within *Calanus* spp. and *Acartia* spp., *Pleuromamma* spp., and *Temora* spp. In addition, *Centropages* sp. was significantly different from *Temora* spp. (Fig. 1b). The Faith’s Phylogenetic Diversity (Faith’s_PD) index of CAB was higher in the *Pleuromamma* spp. (50.75, 16.41–73.45), and the CAB of *Temora* spp. had lower Faith’s_PD, (3.59, 2.45–7.26), respectively (Fig. 1c).

The variation in the Faith's_PD index of CAB was assessed using the Kruskal–Wallis test, which revealed that different copepod genera had a highly significant and phylogenetically distinct bacteriobiome (Fig. 1c). Only the CAB within *Acartia* spp. was not significantly different from *Centropages* sp.

### CAB beta diversity

**Figure 2:**
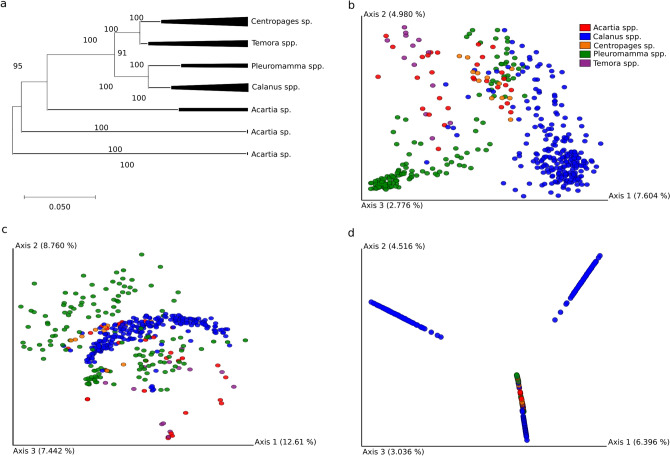
(**a**) 18S rDNA consensus phylogenetic tree of five copepod genera used in the study. (**b**) Unweighted UniFrac distance matrix (community dissimilarity that incorporates phylogenetic relationships between the features); (**c**) Weighted UniFrac distance matrix (community dissimilarity that incorporates phylogenetic relationships between the features); (**d**) Jaccord distance-based beta-diversity. The CAB of representative copepods are colour-coded.

A consensus phylogram of the five copepod genera was constructed (Fig. 2a) (original phylogenetic tree in Fig. S1), and compared with the Unweighted UniFrac distance matrix of CAB using a PCoA plot. In the present study, from the beta-diversity (PERMANOVA P-value 0.001) patterns, phylogenetically closer *Pleuromamma* spp. and *Calanus* spp. harboured CAB expressing a mere 7.604% (axis 1) dissimilarity (Fig. 2b); however, the CAB composition still varied between and within copepod genera. As we closely investigated, Unweighted UniFrac distance matrix showed the CAB of *Pleuromamma* spp. and *Calanus* spp. separated into two different clusters (Fig. 2b), whereas the CAB of *Calanus* spp. was clustered into a single large cluster in a Weighted UniFrac distance matrix (Fig. 2c). In addition, in the Jaccard distance matrix PCoA revealed that *Calanus* spp. had three distinct CAB clusters (Fig. 2d).

On the other hand, the CAB of the phylogenetically closer *Centropages* sp. and *Temora* spp. did show some clustering pattern, but not so distinctive (Fig. 2b).

### Differential abundance of CAB revealed through ANCOM

ANCOM results showed that a total of 23 CAB phyla, viz., Acidobacteria, Actinobacteria, Bacteroidetes, Chlamydiae, Chlorobi, Crenarchaeota, Cyanobacteria, Elusimicrobia, Euryarchaeota, Firmicutes, Fusobacteria, Gemmatimonadetes, GN02, OD1, [Parvarchaeota], Planctomycetes, Proteobacteria, SBR1093, Spirochaetes, [Thermi], TM6, Verrucomicrobia, and WPS-2, were significantly different between copepod genera, with W and Centred Log-Ratio (clr) statistics ranging between 40–30 and 53–2.6, respectively (Table S1). The 23-CAB phyla consisted of 32 classes, 78 orders, 145 families and 240 genera, which were significantly different between copepod genera. From these 240 CAB genera, those in the top two percentile (W and clr statistical values are given in Supplementary File Table S2) were chosen to explain the percentile compositional difference of CAB between copepod genera.

**Figure 3:**
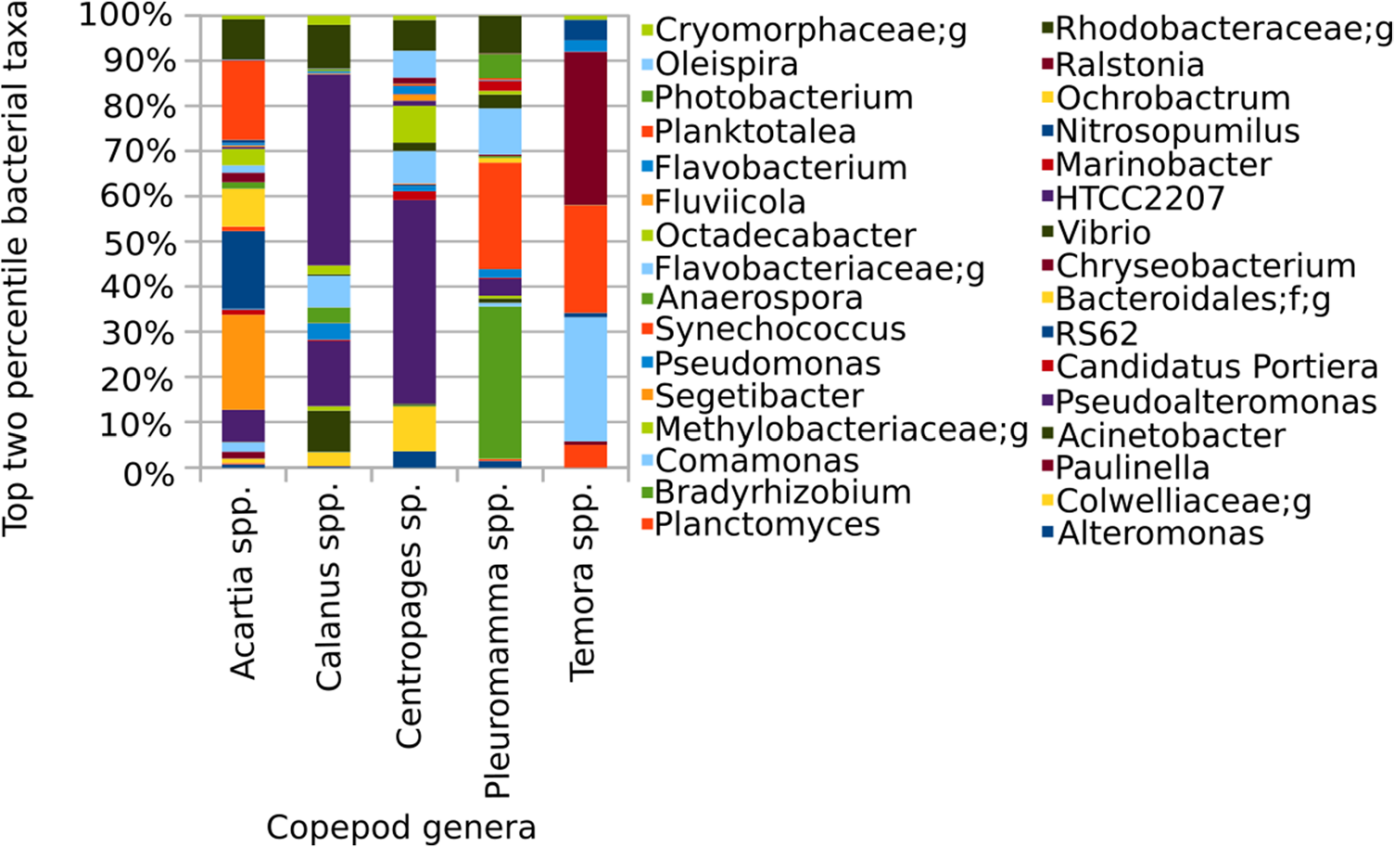
Top two percentages of the CAB-bacterial genera observed in the copepods obtained via ANCOM.

CAB taxa, viz., *Pseudomonas, Anaerospora,* HTCC2207*, Acinetobacter, Ochrobactrum* family Cryomorphaceae, Flavobacteriaceae and Methylobacteriaceae (W and clr-statistical values are given in Supplementary File Table S2) were found in high percentages within *Calanus* spp. (Fig. 3).

Furthermore, from ANCOM, the CAB taxa, viz., *Paulinella,* RS62*, Candidatus Portiera, Planktotalea, Segetibacter, Octadecabacter,* family Rhodobacteraceae and order Bacteroidales, were found in high percentages within *Acartia* spp. (Fig. 3). In the case of *Centropages* sp. the CAB genera, such as *Alteromonas, Pseudoalteromonas, Fluviicola, Oleispira, Ralstonia* and family Colwelliaceae, were found in high percentages. In addition, *Temora* spp. appeared to contain a high percentage of *Comamonas, Planctomyces, Flavobacterium, Synechococcus, Chryseobacterium* and *Nitrosopumilus.* Only four CAB genera, *Bradyrhizobium, Marinobacter, Photobacterium, and Vibrio,* were significantly high in *Pleuromamma* spp. (Fig. 3).

### Machine learning-based models to predict important s-OTUs

**Figure 4:**
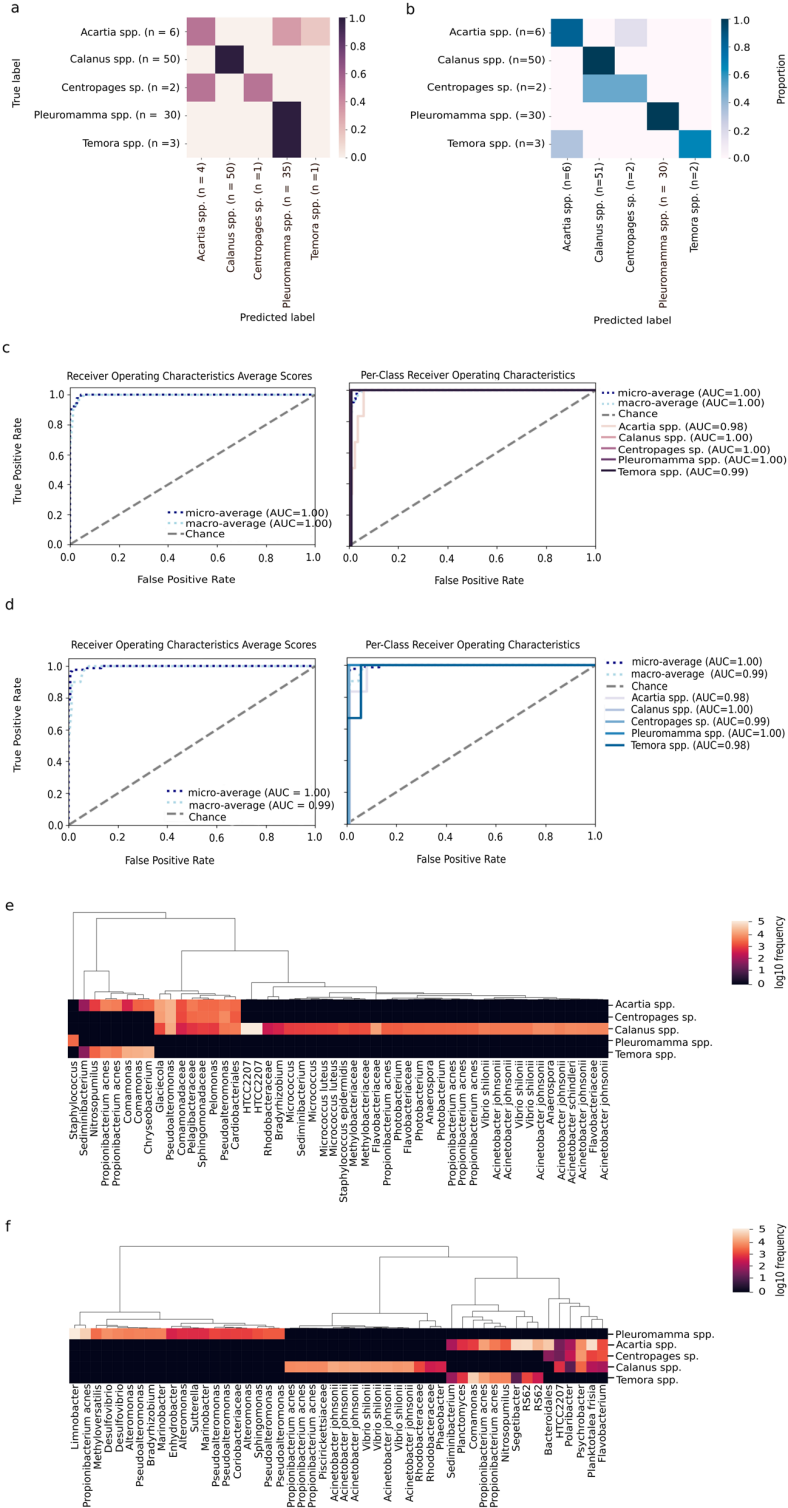
(**a**) Confusion matrix for the RFC model; (**b**) Confusion matrix for the GBC model; (**c**) ROC and AUC for the RFC model; (**d**) ROC and AUC for the GBC model; (**e**) Heatmap of the predicted important s-OTUs in the five copepod genera using RFC; (**f**) Heatmap of the predicted important s-OTUs in the five copepod genera using GBC.

The overall accuracy of the RFC model was 0.923 with an accuracy ratio of 1.68, indicating high reliability (Fig. 4a). However, the GBC model showed better prediction accuracy, with accuracy of 0.967 and an accuracy ratio of 1.76 (Fig. 4b). The accuracy of RFC in predicting important bacterial s-OTUs in copepod genera was within the range of 0.0–1 (Fig. 4a) and the accuracy of GBC in predicting important s-OTUs in the copepod genera was in the range of 0.5–1 (Fig. 4b). The prediction accuracy of important s-OTUs predicted in *Calanus* spp. and *Pleuromamma* spp. by both supervised machine learning (SML) (RFC and GBC) classifiers was high (1.00), unlike the prediction accuracy for *Acartia* spp. (0.5 in RFC and 0.83 in GBC), *Temora* spp. (0.0 in RFC and 0.66 in GBC) and *Centropages* sp. (0.5 in RFC and 0.5 in GBC). The graphical representation of the machine learning model’s Receiver Operating Characteristic (ROC) curve was within the range of 0.98–1 for both RFC and GBC (Fig. 4c,d). This shows the high positive prediction rate and low false prediction rate for both SML classifiers (RFC and GBC).

RFC predicted 25 bacterial taxa and one archaeal taxon in five copepod genera as being important s-OTUs, with differential hierarchical resolutions ranging from the family to species level. From the RFC prediction accuracy values, only the s-OTUs predicted as important s-OTUs for the *Calanus* spp. and *Pleuromamma* spp. are considered, due to the low prediction accuracy for *Acartia* spp., *Temora* spp. and *Cetrophages* sp.. The following s-OTUs were predicted as important by RFC only for *Calanus* spp.: *Photobacterium, Vibrio shilonii, Acinetobacter johnsonii, Acinetobacter schindleri, Micrococcus, Micrococcus luteus*, *Anaerospora,* and Methylobacteriaceae*.* Specific important s-OTUs for the three other genera of copepod were not evident (Fig. 4e).

In the case of GBC, a total of 28 taxa and one archaeal taxon were predicted as important s-OTUs for the five copepod genera (Fig. 4f). From the GBC prediction accuracy values, the only s-OTUs predicted as important were for the *Calanus* spp. and *Pleuromamma* spp., which can further be considered due to the low prediction accuracy for *Acartia* spp., *Temora* spp. and *Centropages* sp., similar to the RFC prediction. The following s-OTUs were predicted as important by GBC only for *Calanus* spp.: *Acinetobacter johnsonii, Vibrio shilonii, Phaeobacter* and Piscirickettsiaceae*.* In *Pleuromamma* spp., s-OTUs of *Marinobacter, Alteromonas, Pseudoalteromonas, Desulfovibrio*, *Limnobacter, Sphingomonas, Methyloversatilis, Enhydrobacter* and Coriobacteriaceae were predicted as important.

### Principal component analysis reveals that copepod genera host functionally distinct bacterial diversity

**Figure 5:**
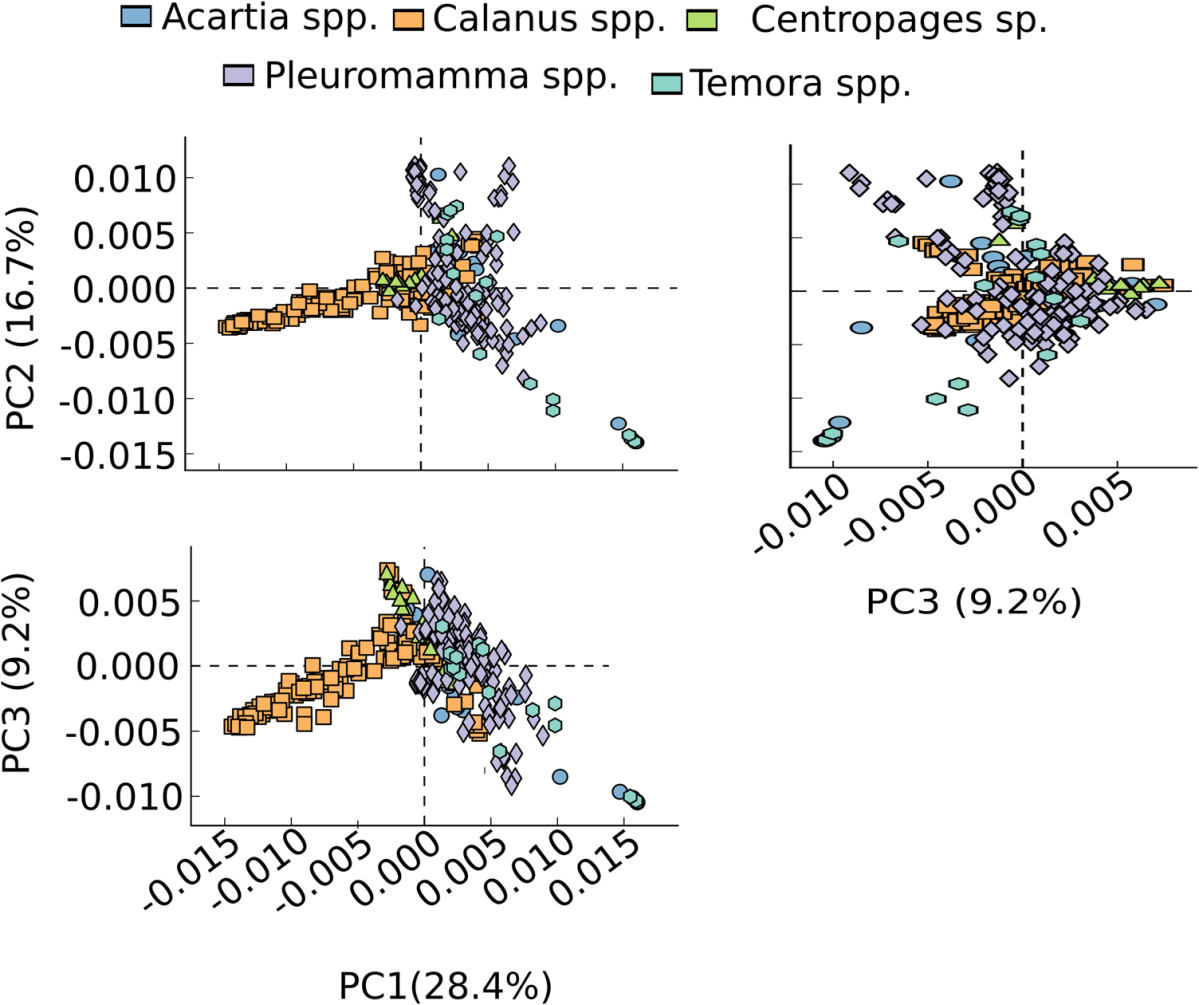
PCA plot for overall diversity pattern of potential functional genes observed among the CAB within the five copepod genera.

From the PCA plot on the potential functional genes of CAB, clusters were found for three copepod genera: *Calanus* spp., *Pleuromamma* spp. and *Centropages* sp. (Fig. 5). The potential functional genes of CAB within *Calanus* spp. clustered from the rest of the copepod genera, with Principal Component (PC) values of 28.4% in axis 1 and 16.7% in axis 2, whereas the potential functional genes of CAB within *Pleuromamma* spp. showed variations of 28.4% in axis PC1 and 9.2% in axis PC3. *Centropages* sp. had unique CAB functional diversity, with variations of 28.4% in axis PC1 and 9.2% in axis PC3, whereas the potential functional genes of CAB within *Acartia* spp. and *Temora* spp. were scattered.

### Biogeochemical potentials of CAB

#### Potential methanogenesis by CAB: evidence of interlinking methanogenesis, DMSP degradation and phosphate utilisation

The genes responsible for the reduction of methyl phosphonate into methane (MPn genes -phnL, phnM, phnJ, phnI, phnH and phnG) were relatively high in the CAB of *Pleuromamma* spp. and *Calanus* spp. (Fig. S2). In addition, based on the present analysis, the CAB of the *Centropages* sp. had the highest proportion of mttB genes, followed by *Acartia* spp. and *Calanus* spp.. One should note that these mttB genes are involved in the oxidation of trimethylamine (TMA) to methyl-CoM (Fig. S2).

CAB of *Pleuromamma* spp. and *Calanus* spp. contained some proportion of dmd-tmd (tri/dimethylation to methylamine) genes, whereas there was little or no proportion of this gene in the CAB of *Temora* spp., *Centropages* sp. and *Acartia* spp. (Fig. S2). The proportion of DmmD gene was highest in the CAB of *Centropages* sp., followed by *Acartia* spp. and *Temora* spp., while the CAB of *Pleuromamma* spp. had the lowest proportion. The proportion of dmdA (DMSP to 3-(methylthio)-propanoate) gene was found to be highest in the CAB of *Acartia* spp. followed by *Centropages* sp. and *Calanus* spp., whereas the proportion was lowest in the CAB of *Temora* spp..

In addition, the dddL gene (DMSP to methyl thioether) was found to be high in the CAB of *Centropages* sp. and *Acartia* spp. and low in the CAB of *Temora* spp.. However, the dmsA gene which converts dimethyl sulfoxide (DMSO) to methyl thioether was found to be highest in the CAB of *Pleuromamma* spp. followed by *Centropages* sp., whereas the genes (dmsB-K00184 and dmsC-K00185) responsible for aerobic conversion of DMSO to methyl thioether were higher in proportion than the anaerobic genes (dmsA-K07306, dmaB-k07307 and dmsC-K07308), which perform the same conversion. The genes dmsB and dmsC (aerobic pathway) were highest in the CAB of *Temora* spp. followed by *Acartia* spp., whereas the dmsB and dmsC (anaerobic pathway) genes were highest in the CAB of *Pleuromamma* spp. followed by *Centropages* sp.

In addition, the dmoA gene which converts methyl thioether to methanethiol was found only in the *Pleuromamma* spp., but in low proportion. Most importantly, mtsA and mtsB genes (which convert methanethiol to methyl-CoM) were found to be high in the CAB of *Pleuromamma* spp., compared to the other copepod genera. Furthermore, the gene responsible for methanogenesis, *i.e.*, the mcrA gene which converts methyl-CoM to CH_4,_ was found to be high in number within the CAB of *Pleuromamma* spp., but in low overall proportion (Fig. S2).

#### Methanotrophic potential of CAB

In the present investigation, we found that the relative abundances of mxaF and mxaI genes responsible for methanol dehydrogenases were high in the CAB of *Pleuromamma* spp. with respect to the CAB of other copepod genera (Fig. S2). Despite a lack of evidence for complete CH_4_ utilisation, the CAB of *Pleuromamma* spp. had the highest proportion of potential genes responsible for the production of methanol dehydrogenase, followed by the CAB of *Centropages* sp. and *Calanus* spp..

### Assimilatory sulphate reduction

Based on our analysis, in all copepod genera assimilatory sulphate reduction (ASR) pathway genes were predominant, rather than the dissimilatory sulphate reduction (DSR) pathway genes. CAB of *Temora* spp. contained a higher number of sulphite reductase ferredoxin components (Fig. S3a), whereas CAB of *Centropages* sp. contained flavoprotein sulphite reductase genes in high proportion (Fig. S3b).

### Nitrogen fixation

The CAB of the copepod genera was screened for the nifH, nifD and nifK genes responsible for nitrogen fixation. CAB of *Pleuromamma* spp. had the highest proportion of nifH gene followed by *Calanus* spp. whereas *Temora* spp. had a lower proportion (Fig. S4).

### Denitrification

Genes involving in all steps of denitrification (nitrate reductions [narG, napA and napB], nitrite reduction [nirK and nirS], nitric oxide reduction [norB, C] and nitrous oxide reduction [nosZ]) were observed in the CAB of all five copepod genera; however, their relative proportions varied between genera. The CAB of *Temora* spp. was found to have the highest proportion of potential denitrification genes, especially narG, napA and napB genes, followed by the CAB of *Pleuromamma* spp., *Centropages* sp., *Calanus* spp. and *Acartia* spp. (Fig. S4).

Among the potential nitrite reductase genes, the proportion of nirK gene was higher than the nirS gene in the CAB of all copepod genera (Fig. S4). Furthermore, the proportion of nirK gene was high in the CAB of *Temora* spp. and *Acartia* spp., whereas a high proportion of nirS was found in *Pleuromamma* spp. and *Calanus* spp. (Fig. S4).

The next step in denitrification is the reduction of nitric oxide to nitrous oxide by norB and norC genes. From the present analysis, we observed that the CAB of *Temora* spp. had the highest proportion of norB gene followed by *Acartia* spp., while the proportion was lowest in *Pleuromamma* spp. followed by *Calanus* spp. and very low in *Centropages* sp. (Fig. S4). In contrast, the gene norC was found highest in *Pleuromamma* spp. followed by *Calanus* spp., and low in *Temora* spp. (Fig. S4). The final reaction is denitrification, *i.e.*, reduction of nitrous oxide to nitrogen by nosZ gene. The CAB of *Acartia* spp. followed by *Calanus* spp. contained a high proportion of nosZ gene (Fig. S4).

#### Anaerobic nitric oxide reduction

The norV (anaerobic nitric oxide reductase) and norW (flavorubredoxin reductase) gene proportions were high in the CAB of *Pleuromamma* spp., compared to in that of (descending order) *Centropages* sp., *Acartia* spp., *Calanus* spp. and *Temora* spp. (Fig. S4).

#### Dissimilatory nitrate reduction into ammonia

The nrfA gene involves in the final step of dissimilatory nitrate reduction into ammonia (DNRA), *i.e.*, reduction of nitrite to ammonia was higher in the CAB of *Calanus* spp., whereas the CAB of *Pleuromamma* spp. and *Centropages* sp. had almost similar proportions of this gene (Fig. S4).

### Carbon processes

The phosphoenolpyruvate carboxylase (ppc) gene is involved in carbon fixation in prokaryotes. This gene was comparatively similar to the other bio-geochemical genes observed in the CAB. While the CAB of *Centropages* sp. had a high proportion of the ppc gene, the CAB of *Pleuromamma* spp., *Temora* spp., *Acartia* spp. and *Calanus* spp. had proportions in descending order (Fig. S5a). In addition, the CAB of *Centropages* sp. had a high proportion of chitinase gene [EC:3.2.1.14], with the least observed in the CAB of *Calanus* spp. (Fig. S5b).

### Role of CAB in iron remineralization

The sequence analysis of CAB showed that the five copepod genera had different proportions of the feoA gene, responsible for ferrous iron transport protein A. The CAB of *Temora* spp. have the highest proportion of feoA gene, followed by *Pleuromamma* spp., *Acartia* spp. and *Calanus* spp. (Fig. S6a). The other gene (fhuF) involved in ferric iron reduction was found to be high in the CAB of *Pleuromamma* spp. (Fig. S6b).

### CAB as a source of cyanocobalamin synthesising prokaryotes

Among the CAB of the five copepod genera analysed, the relative proportion of potential cobalamin-synthesising gene in copepod genera descended in the following order: *Temora* spp., *Acartia* spp., *Calanus* spp., *Pleuromamma* spp., and *Centropages* sp. (Fig. S7). However, from the present study, high proportions of cobalamin-synthesising genes in the CAB of *Temora* spp. may be due to the presence of genus *Nitrosopumilus* (phyla Thaumarchaeota). We found that the CAB may also be one of the potential sources of cyanocobalamin production in the ocean. The limitation of the present study could be the fact that all the CAB sequences were from the Atlantic Ocean.

## Discussion

### CAB diversity between the copepod genera

*Calanus* spp. are filter feeders and mostly herbivores, but do feed on ciliates and other heterotrophic protists during reproduction and energy shortfall^38,39^. This may be the reason for their high H index. Most of the gene sequences used for this meta-analysis were from *Calanus finmarchicus*; however, *Centropages* sp. feeds on different sources, from microalgae to fish larvae^40^. *Acartia* spp. are primarily omnivorous (with a high degree of carnivore behaviour), feeding on phytoplankton, rotifers, and occasionally ciliates^41^, whereas *Temora* spp. frequently switches its feeding behaviour, *i.e.*, from omnivore to herbivore, based on season and on food availability^42^. The bacterial alpha diversity analysis in the *Temora* spp. revealed a significantly lower Shannon diversity. However, in an earlier study, no difference was reported in alpha diversity between the *Temora* sp. and *Acartia* sp.^37^. This can be explained based on the source of copepods involved for the study by Wega et al.^37^, which was based only on a single source, *i.e.*, the central Baltic sea; however, in our case the CAB sequences for *Acartia* spp. were from the central Baltic sea^37^ as well as the Gulf of Maine^10^. The occurrence of high Faith’s_PD in *Pleuromamma* spp. may be due to their range distribution in the water column, and few species within *Pleuromamma* spp. are known to migrate vertically^11,43^, or possibly due to their food uptake, which includes phytoplankton, microzooplankton (ciliates and flagellates) and detritus^11,44^.

The consensus phylogram revealed that, at the genera level, *Calanus* spp. was phylogenetically closer to *Pleuromamma* spp. and formed two distinct clusters in the PCoA plot. Furthermore, the difference in dissimilarity percentage of CAB between *Pleuromamma* spp. and *Calanus* spp. may be attributed to the difference in vertical migration, life stages and feeding behaviour between the two copepod genera. *Pleuromamma* spp., an omnivorous feeder^11,44^, can migrate vertically up to 1000 m^11,43^ whereas *Calanus* sp., mostly herbivores but occasional omnivores^36,37^, can migrate up to 600 m^45,46^. This may also be due to the difference in the life stage of *Calanus* sp. (the microbial communities varied between diapausing and active feeding)^2^.

### ANCOM

In an early report, bacterial members belonging to the Gammaproteobacteria were observed to be dominant in *Calanus finmarchicus*, followed by members of Alphaproteobacteria^10^. However, in the present ANCOM, the presence of Gamma and Alphaproteobacteria were equal (three genera each) in *Calanus* spp*.* (Fig. 3). Similar to our results, the unclassified genus of Rhodobacteraceae was reported to be abundant in *Acartia longiremis*^10^. Colwelliaceae was reported to be abundant in *Calanus finmarchicus*^10^; however, in the present analysis, family Colwelliaceae was found in a high percentage in *Centropages* sp.. An abundance of Flavobacteriaceae was observed, along with phytoplankton and diatoms in the gut of *Calanus finmarchicus* containing food^2^, whereas *Sedinimicola* sp. (Flavobacteriaceae) was observed to be dominant in *Acartia longiremis*, *Calanus finmarchicus* and *Centropages hamatus*^10^. In addition, Dorosz et al.^47^ reported that *Flavobacterium* was more dominant in *Temora longicornis* than in *Acartia tonsa*, whereas, in our case, Flavobacteriaceae was found in a high percentage in *Calanus* spp.. Upon comparison of the present ANCOM and previous reports, *Pseudoalteromonas* sp. appeared in high percentage not only within *Centropages* sp.^10^ but also in consistent and abundant bacteria in *Acartia* sp., and *Calanus* sp. The prevalence of *Pseudomonas* has been observed in *Pleuromamma* sp.^11^, whereas this was not the case in our analysis (Fig. 3). Similarly, Cregeen^11^ analysed the bacteriobiome of *Pleuromamma* sp. and observed the dominance of *Alteromonas*, but, from our meta-analysis, a higher abundance of *Alteromonas* was observed in *Centropages* sp. compared to five other genera, including *Pleuromamma* spp. (Fig. 3).

From our analysis, *Nitrosopumilus* was observed contain a high amount of *Temora* spp., but the abundance of *Nitrosopumilus* was reported to show no difference between the particle-associated in the water column and within *Temora* sp.^37^; thus, the high percentage observed in our analysis may be due to the exchange of *Nitrosopumilus* from seawater. Vibrionales was identified as a core member in the gut of *Pleuromamma* spp.^1^, similar to the present analysis, wherein *Vibiro* percentage was found to be high in the CAB of *Pleuromamma* spp.. The copepods were reported to have a selective niche of *Vibrio* capable of degrading chitin^1,48^. In the present analysis, seven bacterial taxa were found to be in high percentages in *Centropages* sp. and, among those seven, four taxa belong to the Gammaproteobacteria. A high proportion of Gammaproteobacteria in *Centropages* sp. was also reported previously^10^.

### Machine learning-based prediction

The masking effect of the abundant bacterial community associated with the copepod diet and ambient water column should not hinder the detection of core OTUs, as evidenced by previous studies^1,2^. QIIME2 core_abundance algorithms used in the present study did not predict single bacterial s-OTUs (data not presented). Hence, we used machine learning approaches to detect important core s-OTUs specific to copepod genera.

From our SML classifier results, the important s-OTUs predicted in *Calanus* spp. and *Pleuromamma* spp. were found to have high prediction accuracy (area under the curve (AUC) = 1.00). Therefore, we discuss the important s-OTUs predicted for these two copepod genera (*Calanus* spp. and *Pleuromamma* spp.). To begin with, among the important s-OTUs predicted in *Calanus* spp. from the present analysis (both SML models: RFC and GBC), Gammaproteobacteria was a dominant member (15 and 9 s-OTUs from RFC and GBC, respectively) followed by Alphaproteobacteria, which represents 6 and 3 s-OTUs from RFC and GBC, respectively. This observation was similar to that in an earlier study, where Gammaproteobacteria and Alphaproteobacteria were reported as core OTUs in *Calanus finmarchicus*^2^. In addition, within the Gammaproteobacteria, seven (RFC) and five (GBC) s-OTUs representing the *Acinetobacter* (Moraxellaceae) were predicted as important s-OTUs in the present study, similar to an earlier study in which Moraxellaceae was reported to be closely associated with *Calanus finmarchicus*^10^. Moreover, four s-OTUs of *Acinetobacter* (Moraxellaceae) were also reported as core OTUs in *Calanus finmarchicus*^2^. In addition to the present analysis, three s-OTUs from both SML classifiers (RFC and GBC) belonging to *Vibrio shilonii* were predicted as important s-OTUs in *Calanus* spp.. Comparably, four OTUs of Vibrionaceae (three OTUs of *Vibrio* sp. and one similar to *Vibrio harveyi*) were observed in *Calanus finmarchicus*^2^.

In the present SML analysis, one genus *Bradyrhizobium* (order Rhizobiales), was predicted as an important s-OTU in *Pleuromamma* spp. by GBC classifiers. Moreover, in the present ANCOM, *Bradyrhizobium* was found in a high percentage within *Pleuromamma* spp.. This *Bradyrhizobium* is also known to contain nifH gene, as they usually occur in seawater^49^ and SML-GBC also predicted this genus as an important s-OTU in *Calanus* spp.. Bradyrhizobiaceae was also found to be the most abundant OTU, contained in 79 of the total 137 sequences in the negative control in a similar analysis^1^. Thus, in the case of *Bradyrhizobium,* a further investigation is required in order to come to a meaningful conclusion.

Moreover, in a previous study, order Vibrionales was also predicted as a core member (based on presence/absence) in *Pleuromamma* spp.^1^. The genus *Pseudoalteromonas* was also already reported as occurring in high abundance in *Pleuromamma* sp.^11^. However, in the present analysis, GBC predicted five s-OTUs of *Pseudoalteromonas* as important s-OTUs in *Pleuromamma* spp., whereas RFC predicted two s-OTUs of *Pseudoalteromonas* as important s-OTUs in *Acartia* spp., *Calanus* spp., and *Centropages* sp. (Fig. 4e). This is similar to *Pseudoalteromonas*, which is reported as a constant and stable OTU in *Acartia* sp.^37^, *Calanus* sp.^2^ and *Centropages* sp.^10^. Thus, it is unwise to consider *Pseudoaltermonas* as being specific to one copepod genera.

In the present study, the GBC model predicted three s-OTUs of *Alteromonas* and two s-OTUs of *Marinobacter* as important ones in *Pleuromamma* spp., and ANCOM also showed that the genus *Marinobacter* proportion was high in *Pleuromamma* spp.. Comparably, both *Alteromonas* and *Marinobacter* were reported as common in *Pleuromamma* sp.^11^. Though the abundance of genus *Sphingomonas* was low, it was reported to appear consistently in *Pleuromamma* sp.^11^, and our analysis predicted this genus as an important s-OTU of *Pleuromamma* spp. (from GBC) (Fig. 4f).

In the present study, the GBC model predicted *Limnobacter* as an important s-OTU in *Pleuromamma* spp., and ANCOM also showed that the proportion of genus *Limnobacter* was high in *Pleuromamma* spp.. Moreover, in a previous study, *Limnobacter* was reported to occur in high abundance in, as well as being unique to, copepods (*Pleuromamma* spp.)^11^. Also, the genera *Methyloversatilis* was reported to be low in abundance in *Pleuromamma* spp., whereas the SML-GBC model in this study predicted this genus to be an important s-OTU in *Pleuromamma* spp. (Fig. 4f). The order Pseudomonadales was reported as a core member in *Pleuromamma* spp.^1^; however, our GBC model predicted the bacterial genera *Enhydrobacter* (Pseudomonadales) as an important s-OTU in *Pleuromamma* spp. (Fig. 4f). In addition, from ANCOM, this genus *Enhydrobacter* was found in high percentage in *Pleuromamma* spp., but was also reported to be high in proportion in calanoid copepods^6^. One another important s-OTU predicted in *Pleuromamma* spp. by our GBC model was *Desulfovibrio*, and ANCOM also showed that the proportion of genus *Desulfovibrio* was found to be high in *Pleuromamma* spp..

HTCC2207 (Gammaproteobacteria) was predicted as an important s-OTU in *Calanus* spp. by both SML models. Also, from ANCOM, HTCC2207 was found in a high percentage in *Calanus* spp.. HTCC2207 is usually more abundant in seawater, and has been reported as present in *Acartia longiremis*., *Calanus finmarchicus* and *Centropages hamatus* with a full gut^10^. Due to their known proteorhodopsin gene and being free water—living bacteria^50^, the probability of detecting this bacterium in the copepod gut may be determined by food ingestion.

*Sediminibacterium* (Chitinophagaceae) was reported to be in low abundance but regularly present in *Pleuromamma* sp.^11^. However, in the present analysis, the RFC model predicted *Sediminibacterium* as important s-OTUs in *Acartia* spp., *Calanus* spp. and *Temora* spp. (Fig. 4e,f), whereas the GBC model predicted *Sediminibacterium* as important s-OTUs in *Acartia* spp. and *Temora* spp. (Fig. 4). Chitinophagaceae was reported to be associated with calanoid copepods in the North Atlantic Ocean^6^. Earlier studies showed that the genus *Photobacterium* (Phylum: Proteobacteria) was abundant in *Pleuromamma* sp.^11^, *Centropages* sp.^10^, and *Calanus finmarchicus*^2^. Herein, *Photobacterium* was detected as an important s-OTU in *Calanus* spp. by the RFC model only. Furthermore, in the present analysis, *Nitrosopumilus* was predicted as an important s-OTU in *Acartia* spp. and *Temora* spp. by both the SML models, and this genus was also reported to be in high percentage in *Acartia* sp. and *Temora* sp.^37^.

Furthermore, RFC predicts *Pelomonas* as an important s-OTU in *Acartia* spp., *Centropages* sp. and *Calanus* spp.. However, in a previous study, *Pelomonas* was ruled out as a core OTU in *Calanus* spp.^2^. The GBC predicted two s-OTUs of RS62 and one s-OTUs of *Planctomyces* as important ones in *Acartia* spp., and *Temora* spp.. RS62 belongs to the order Burkholderiales, and though this order was reported to be abundant, abundance varied between individual copepods (*Acartia* sp. and *Temora* sp.)^37^. Burkholderiales was also reported as a main copepod-associated community^9^. However, in the present study, the genus *Comamonas* belonging to Burkholderiales was predicted as an important s-OTU in *Acartia* spp., and *Temora* spp. by both SML models.

Approximately 25 taxa detected by the RFC approach were also found in high percentages from ANCOM. Among them, five s-OTUs, viz*.*, *Anaerospora, Micrococcus, Micrococcus luteus, Vibrio shilonii and* Methylobacteriaceae*,* were predicted as important s-OTUs in *Calanus* spp. in our report, for the first time (Fig. 4e). From the 28 taxa detected by the GBC model, four s-OTUs, viz*.*, *Phaeobacter, Acinetobacter johnsonii, Vibrio shilonii,* and Piscirickettsiaceae, were predicted as important s-OTUs in *Calanus* spp. in our report, for the first time (Fig. 4f). In addition, eight s-OTUs, viz*.*, *Marinobacter*, *Limnobacter. Methyloversatilis, Desulfovibrio, Enhydrobacter, Sphingomonas, Alteromonas* and Coriobacteriaceae*,* were predicted as important s-OTUs in *Pleuromamma* spp. in the GBC model, for the first time.

### Potential biogeochemical genes of CAB and their variation and abundance

Bacterial communities exploit copepods as microhabitat by colonising copepods’ internal and external surfaces, and mediate marine biogeochemical processes^9^. CABs also metabolise organic compounds, such as chitin, taurine, and other complex molecules in and around the copepod, which may be a hotspot for the biogeochemical process^9^. In an earlier analysis, potential functional genes in the water column of the Southern Ocean were processed using Parallel-Meta3 software^51^; herein, we have used a more advanced PICRUSt2 analysis to screen for the potential functional genes.

#### Methanogenesis

In the present analysis, the bacterial taxa involved in methane production, viz. methanogenesis, methylphosphonate, DMSP and DMSO, were observed in all copepod genera but relative proportion varied between genera. A similar observation in *Acartia* sp. and *Temora* sp. has been reported^37^.

In the present analysis, we found that CAB has a complete set of aerobic methanogenesis genes (PhnL, M, J, H and G) which convert methylphosphonate (MPn) to methane (CH_4_)^52^. Some copepods, like *Acartia* sp. and *Temora* sp., were reported to associate with bacteria involved in CH_4_ production from MPn^37^. De Corte et al.^9^ suggested that different copepod species have different CAB, and only some copepods have the specific CAB for methanogenesis and other biogeochemical cycles.

A previous study (with 14 C-labelled experiments) observed high methane production in *Temora longicornis* compared to *Acartia spp.*^53^. In addition, the methanogenic archeae *i.e.*, *Methanobacterium bryantii*-like sequences, *Methanogenium organophilum, Methanolobus vulcani*-like sequences and *Methanogenium organophilum* were noted in *Acartia clausi* and *Temora longicornis* faecal pellets^54^. In the present study, we observed that *Pleuromamma* spp. has a high proportion of the mcrA gene (Fig. S2).

*T. longicornis* fed with a high content of TMA-/DMA-rich phytoplankton produced the maximum amount of CH_4_, suggesting that this production may be due to the micro-niches inside the copepods^55^. However, in our analysis, CAB of *Pleuromamma* spp. was found to have a high proportion of the dmd-tmd gene.

In our meta-analysis, *Acartia* spp. was found to have a high proportion of the dmdA gene. The taxa detected in the present study, such as Pelagibacteraceae, some Alpha and Gammaproteobacteria, are known to have dmdA genes^56^.

Copepods feeding on phytoplankton liberate DMSP, which, in turn, is utilised by the DMSP-consuming bacteria in the gut (*Acartia tonsa*), leading to methane production^57^. Moreover, the methane enrichment in the Central Baltic Sea is due to the dominant zooplankton *Temora longicornis* feeding on the DMSP-/DMSO-rich Dinophyceae, resulting in methane release^53^.

Instead of analysing faecal pellets^57^ and anaerobic incubation experiments^58^, further research should also consider CAB-mediated aerobic methanogenesis as one factor with which to solve the ‘ocean methane paradox’.

##### Methanotrophic potential of CAB

The present analysis showed that the CABs of *Pleuromamma* spp. and *Centropages* sp. were had a high proportion of methanol dehydrogenase genes (mxaF and mxaI) (Fig. S2). This may be due to the presence of Proteobacteria that involves methane oxidation, viz*.*, Beijerinckiaceae, Methylococcaceae, Methylocystaceae and Verrucomicrobia (Supplementary File Table S3)^59^.

#### Assimilatory sulphate reduction

A relative abundance of taxa such as *Synechococcus* and the Deltaproteobacterial family (unclassified genera in Desulfovibrionaceae), Rhodobacteraceae and Flavobacterium (Supplementary File Table S3) were observed in the CAB of *Temora* spp., which may be responsible for the ASR pathway, as these taxa are known to have ferredoxin-sulphite reductase activity (Supplementary File Table S3).

#### Nitrogen fixation

A high abundance of nifH gene was reported in copepods collected from the coastal waters of Denmark (Øresund) (mostly contributed by *Acartia* spp.), with *Vibrio* spp*.* as dominant members^16^. However, in the present study, the nifH gene was found to be high in the CAB of *Pleuromamma* spp. (Fig. S4), and one should note that this may be due to the high abundance of genus *Vibrio* in the CAB of *Pleuromamma* spp. (Supplementary File Table S3). *Vibrio* attached to the exoskeleton and gut lining of copepods^60^ using chitin as both a carbon and energy source was previously reported^10^. Furthermore, copepods are reported to be a hotspot for nitrogen fixation at a rate of 12.9–71.9 μmol N dm^−3^ copepod biomass per day^16^. The abundance of nifH gene in the CAB of *Pleuromamma* spp. may be due to the presence of genera including *Synechococcus, Prochlorococcus, Bradyrhizobium, Microcystis,* and *Trichodesmium* (Supplementary File S3).

##### Denitrification

In our analysis, the CAB of *Temora* spp. were found to have the highest proportion of napA and napB genes (Fig. S4), followed by *Pleuromamma* spp., whereas an abundance of napA and narG genes were reported in North Atlantic copepods contributed by *Calanus* sp. and *Paraeuchaeate* sp.^9^. However, in the present analysis, the CAB of *Temora* spp. was found to have a high proportion of narG (Fig. S4). Bacterial genera including *Pseudoalteromonas, Actinobacterium* and *Shewanella* also contain the nirS gene, as reported in both live and dead *Calanus finmarchicus*^14^. Likewise, from our analysis, both *Pseudoalteromonas* and Actinobacteria were found in *Calanus* spp.. A metagenome analysis of copepod-associated microbial community reported them having genes responsible for denitrification and DNRA^9^.

##### Anaerobic nitric oxide reduction

Families including Aeromonadaceae and Enterobacteriaceae were observed in the CAB of *Pleuromamma* spp. and *Calanus* spp., in relatively higher proportion than in other copepods. The genera *Aeromonas* (family Aeromonadaceae)^61^ and *Escherichia coli* (family Enterobacteriaceae)^62^ are known to contain norV genes. The presence of these bacterial taxa in *Pleuromamma* spp. may be due to feeding of ciliates, flagellates, and detritus particles^11,44^. This may be one reason for a high proportion of norV and norW genes in these copepods (Fig. S4).

#### Carbon processes

**Figure 6:**
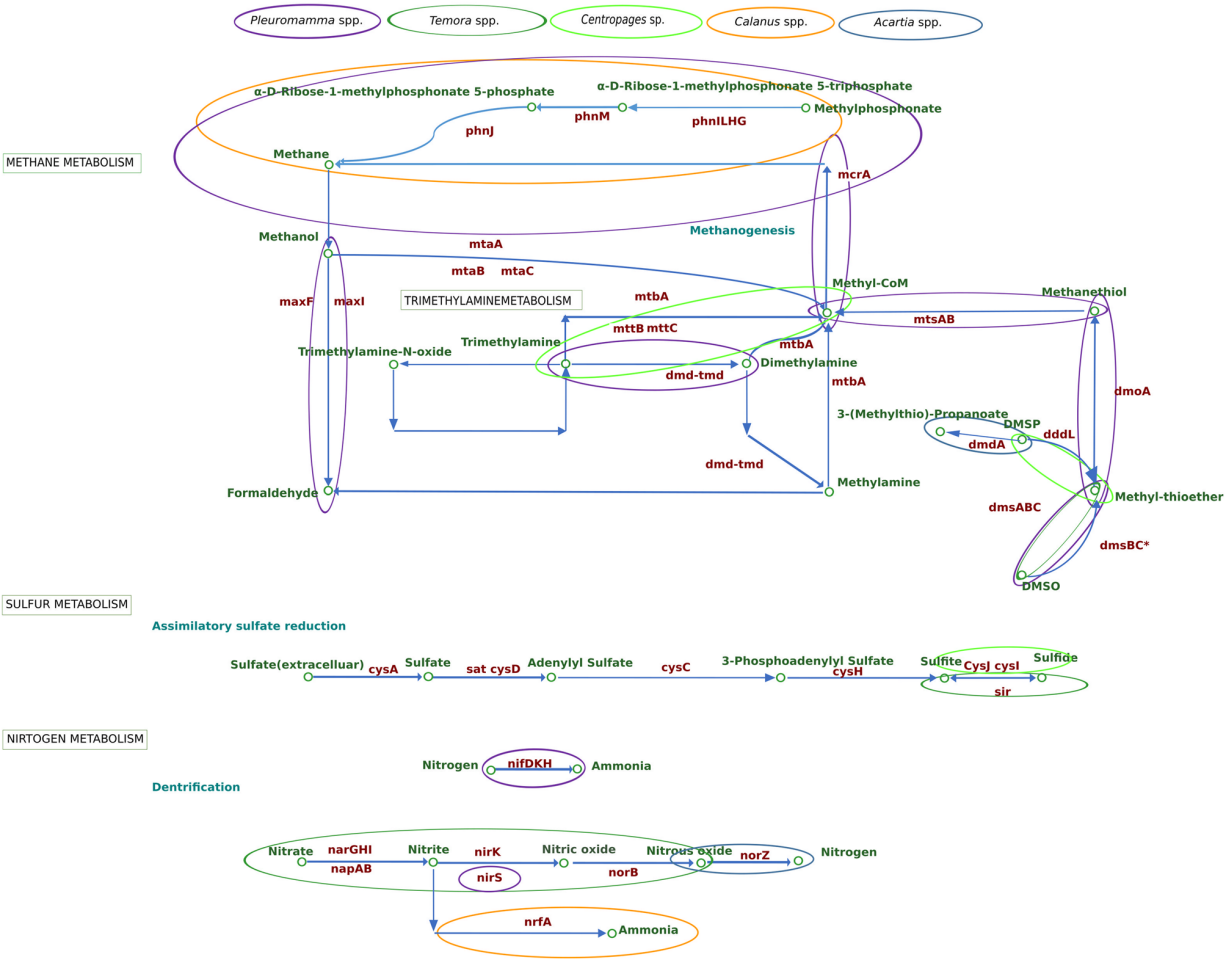
Overall representation of the potential functional genes of CAB involved in biogeochemical cycles. The circle and colour represent the copepod genera contained in high proportion for that particular biogeochemical process.

Bacterial taxa like Colwelliaceae^10,63^ *Flavobacterium, Arthrobacter, Serratia, Bacillus, Enterobacter, Vibrio*^64^, *Pseudoalteromonas*^63^ and *Achromobacter*^65^ produce chitinase. The presence of chitinase gene in CAB is unsurprising, as their foregut and hindgut are both made up of chitin^11^. The overall outline of CAB-mediated biogeochemical pathways is represented in Fig. 6.

#### Role of CAB in iron remineralization

*Pleuromamma* spp. carries a similar proportion of ferric iron reductase (fhuF) and ferrous iron transport protein A (feoA) genes (Fig. S6a,b). The presence of a high proportion of ferric iron reductase gene fhuF in *Pleuromamma* spp. requires detailed investigation. It was reported that acidic and low-oxygen conditions in the copepod gut may assist iron dissolution and remineralisation, forming soluble Fe(II)^13,66^. This increases the iron bioavailability in the surroundings, promoting phytoplankton growth^66^. In addition, bacterial community associated with the zooplankton, such as Bacteroidetes, Alphaproteobacteria and Gammaproteobacteria, are known to carry genes involved in iron metabolism^9^.

In an early study on *Thalassiosira pseudonana* fed to *Acartia tonsa*, iron was found in the faecal pellets^67^. However, in the present analysis, *Acartia* spp. was found to have a lower proportion of the feoA gene compared to *Temora* spp. and *Pleuromamma* spp.. Moreover, genes involved in iron metabolism were reported to be high in zooplankton-associated microbiome^9^.

The differential iron contributions of different copepod genera were unknown until now. For organisms that must combat oxygen limitation for their survival (*Pleuromamma* spp.), pathways for the uptake of ferrous iron are essential. Nevertheless, the meta-analysis performed here showed that *Pleuromamma* spp. may be a significant contributor to both iron bioavailability and nitrogen fixation.

#### CAB as a source of cyanocobalamin-synthesising prokaryotes

Organisms within all domains of life require the cofactor cobalamin (vitamin B12), which is usually produced only by a subset of bacteria and archaea^68^. Previous studies reported that the cobalamin in ocean surface water is due to de novo synthesis by Thaumarchaeota. Moreover, few members of Alphaproteobacteria, Gammaproteobacteria and Bacteroidetes genomes were reported to contain the cobalamin-synthesising gene^68^. In our analysis, the CAB of *Temora* spp. was found to have a high proportion of Thaumarchaeota, whereas Alpha-gammaproteobacteria content was found to be high in the CAB of *Acartia* spp., *Calanus* spp. and *Pleuromamma* spp.. In this regard, further studies on CAB diversity from different ocean realms would shine a light on the actual potential of CAB in global biogeochemical cycles.

## Conclusion

Herein, five copepod genera, viz*.*, *Acartia* spp., *Calanus* spp., *Centropages* sp., *Pleuromamma* spp., and *Temora* spp., and their associated bacteriobiomes were investigated. The use of meta-analysis in the present study reveals the difference in bacterial diversity indices within the alpha and beta-diversity. To be more specific, the meta-analysis showed significant variations in the alpha diversity between the copepod genera. Moreover, it was revealed that *Calanus* spp. have high Shannon index (H-index), and *Pleuromamma* spp. have high Faith’s Phylogenetic Diversity. Furthermore, the meta-analysis revealed that the CAB within the phylogenetically closer *Pleuromamma* spp. and *Calanus* spp. expressed a mere 7.604% (axis 1) dissimilarity distance in PCoA analysis (Unweighted UniFrac distance matrix, based on the phylogenetic index). Likewise, from the meta-analysis, we were able to identify the bacterial taxa which are significantly abundant in each copepod genera in comparison with others.

In earlier studies, the core bacterial OTUs were identified based on their presence/absence^1^, as well as by using distribution-based clustering (DBC) algorithms^2^. Herein, machine learning models were used to predict the important copepod-associated bacterial genera within the five different copepod genera. In specific, we used supervised machine learning models to predict the important bacterial s-OTUs. We predicted 28 bacterial taxa and one archeael taxon (SML-GBC) as important s-OTUs in the five copepod genera. Among the predicted bacterial genera and families, *Vibrio shilonii, Acinetobacter johnsonii, Phaeobacter* and Piscirickettsiaceae were reported as common important s-OTUs in the *Calanus* spp. and *Marinobacter, Limnobacter. Methyloversatilis, Desulfovibrio, Enhydrobacter, Sphingomonas, Alteromonas* and Coriobacteriaceae were predicted as important s-OTUs in *Pleuromamma* spp., for the first time. Additionally, the prediction accuracy (for *Calanus* spp. and *Pleuromamma* spp.) of the machine learning models used here showed high accuracy, indicative of the reliability of the predicted important s-OTUs in the copepod genera. Notably, from the machine learning-based classification it was evident that specific bacterial s-OTUs do exist for copepods.

Furthermore, our meta-analysis revealed that the five copepod genera have bacterial communities that are capable of mediating methanogenesis (with evidence of interlinking of methane production, DMSP degradation and phosphate utilisation) and methane oxidation. We also found the five copepod genera to have more potential ASR microbial communities than DSR communities within the CAB. Likewise, bacterial communities with potential genes involved in nitrogen fixation, denitrification and DNRA were also observed among the CAB of these five copepod genera. We also found the potential genes that perform carbon fixation, iron remineralisation and cyanocobalamin (vitamin B12) synthesis in the CAB of the five copepod genera.

## Supplementary Information

Supplementary Information 1.
