# Supplementary material for: Meta-analysis cum machine learning approaches address the structure and biogeochemical potential of marine copepod associated bacteriobiomes
Source: Sci Rep. 2021 Feb 8;11:3312. doi: 10.1038/s41598-021-82482-z (PMC7870966; doi:10.1038/s41598-021-82482-z)
Supplement: Supplementary file 1 — Supplementary Information 1. [file 41598_2021_82482_MOESM1_ESM.docx]

**Meta-analysis cum machine learning approaches address the structure and biogeochemical potential of marine copepods associated bacteriobiome**

Balamurugan Sadaiappan^1, +^, PrasannaKumar Chinnamani^1, +^, Uthara V Nambiar^1^, Mahendran Subramanian^2, 3^, Manguesh U Gauns^1, *^

Plankton Ecology Lab, Biological Oceanography Division, CSIR-National Institute of Oceanography, Dona Paula, Panaji-403004, Goa, India.

^2^Department of Biphosphoenolpyruvate carboxylaseoengineering and Department of Computing, Imperial College London, South Kensington-SW72AZ, London, United Kingdom.

^3^Faraday-Fleming Laboratory, W148TL, London, United Kingdom.

Corresponding author: Manguesh U Gauns (gmangesh[@nio.org)](mailto:email@address.edu))

**Contents of this file**

Figures S1 to S7

Tables S1

**Additional Supporting Information (Files uploaded separately)**

Legends for Excel files S1 and S2.

**Introduction**

The following methods were used to derive the supplementary figures provided in this file.

Phylogenetic tree of copepods was drawn using Mega 10. (Supplementary Figure S1).

For identifying potential functional genes associated with the CAB was computed using Phylogenetic Investigation of Communities by Reconstruction of Unobserved States (PICRUSt2) (Douglas et al., 2020). The output abundance KEGG data were analyzed in the Statistical Analysis of Metagenomic Profiles (STAMP) which includes Principle Component Analysis (PCA) (Parks et al., 2014). Furthermore, the heatmap and differential abundance in the functional gene was plotted using STAMP. (Supplementary Figures S2 and S7).

**Supplementary Figures**


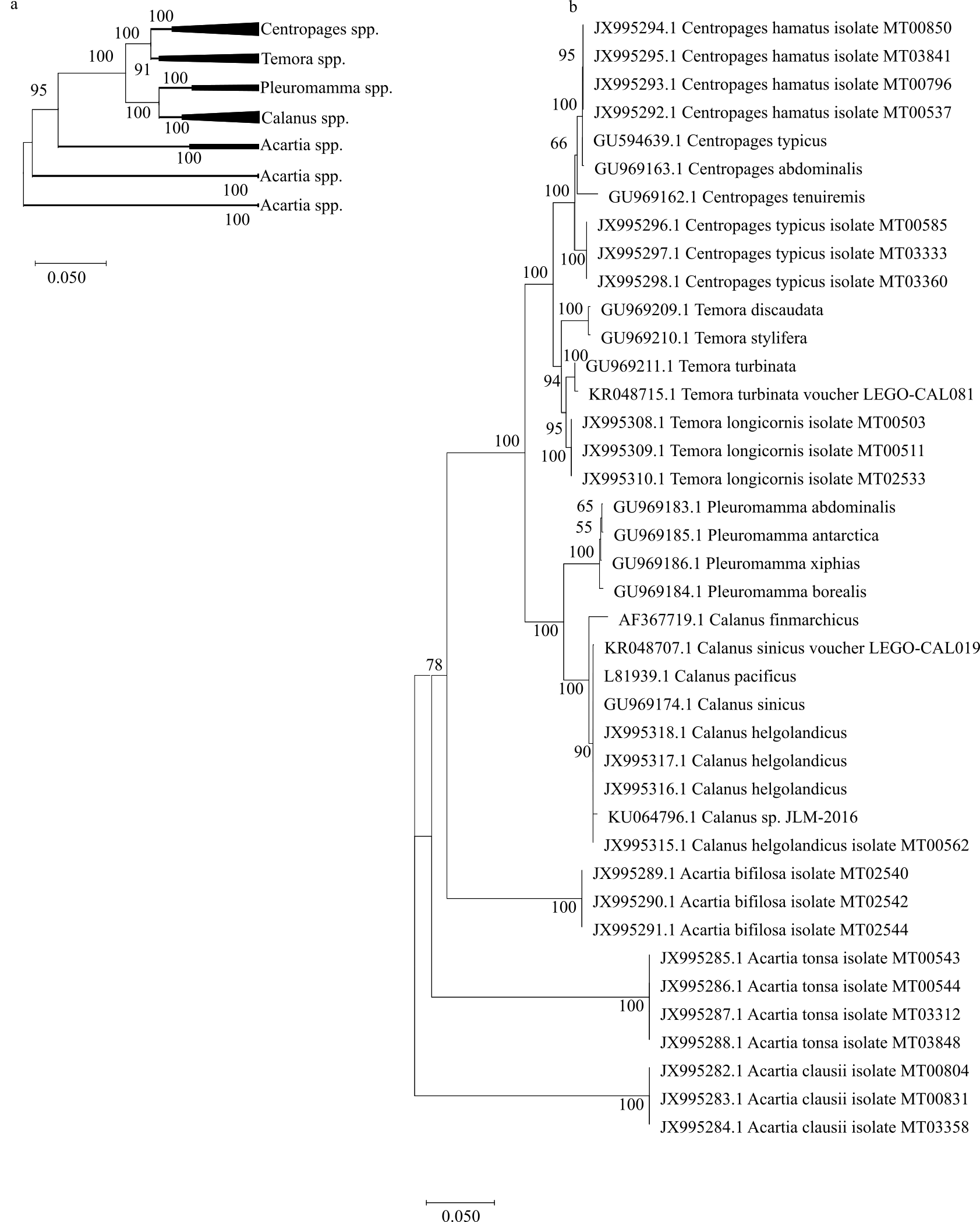


Figure S1. Neighbor joining phylogenetic tree (18S rDNA) of five copepod genera constructed from 10, 7, 4, 9 and 10, species of *Centropages* sp., *Temora* spp., *Pleuromamma* spp., *Calanus* spp., and *Acartia* spp., sequences respectively.


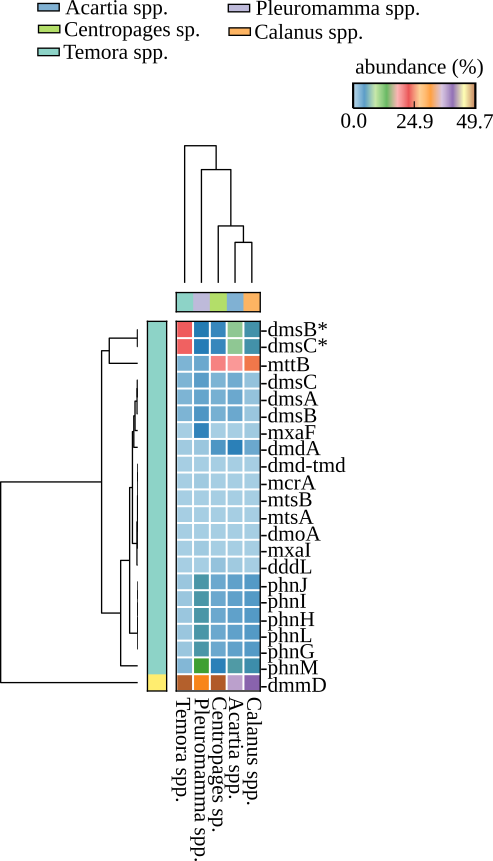


Figure S2. The heatmap represents the relative proportion of methanogenesis and methanotrophic genes observed in CAB of five copepods genera with gene name.


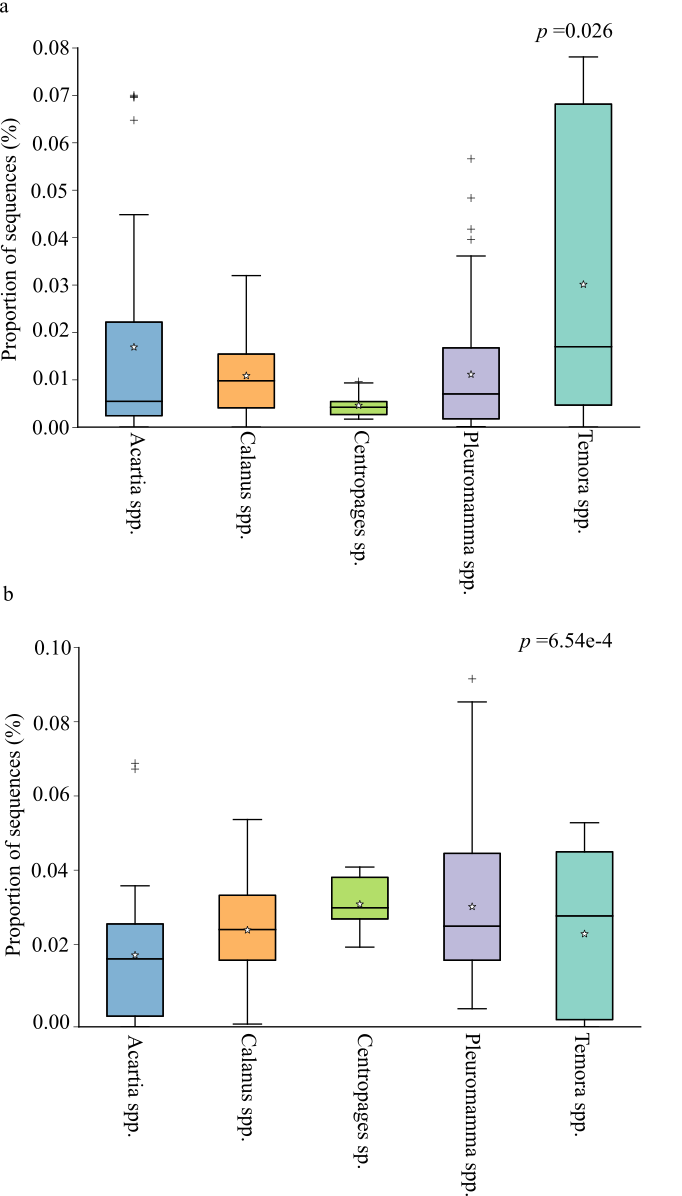


Figure S3. The relative abundance of a) Sulphite reductase (ferredoxin) b) Sulphite reductase (NADPH) flavoprotein alpha-component in CAB of copepods genera.

***
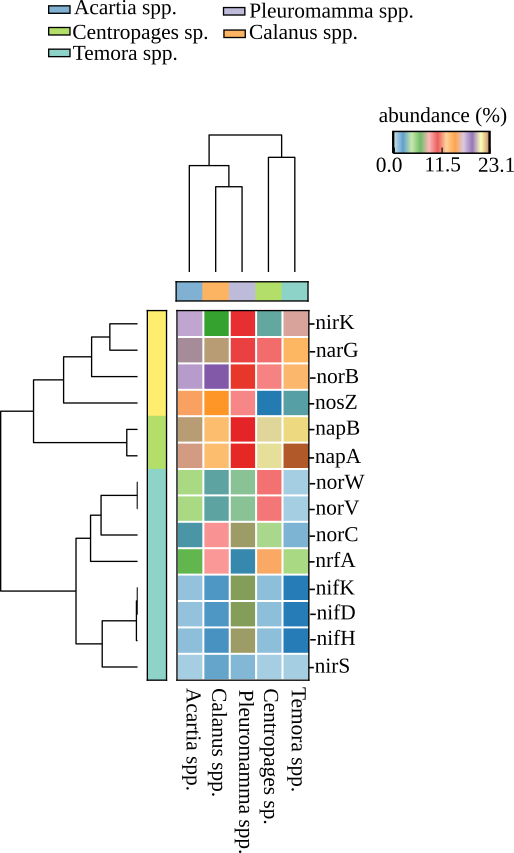
***

Figure S4. The heatmap represents the relative proportion of nitrogen cycle genes observed in CAB of five copepods genera.


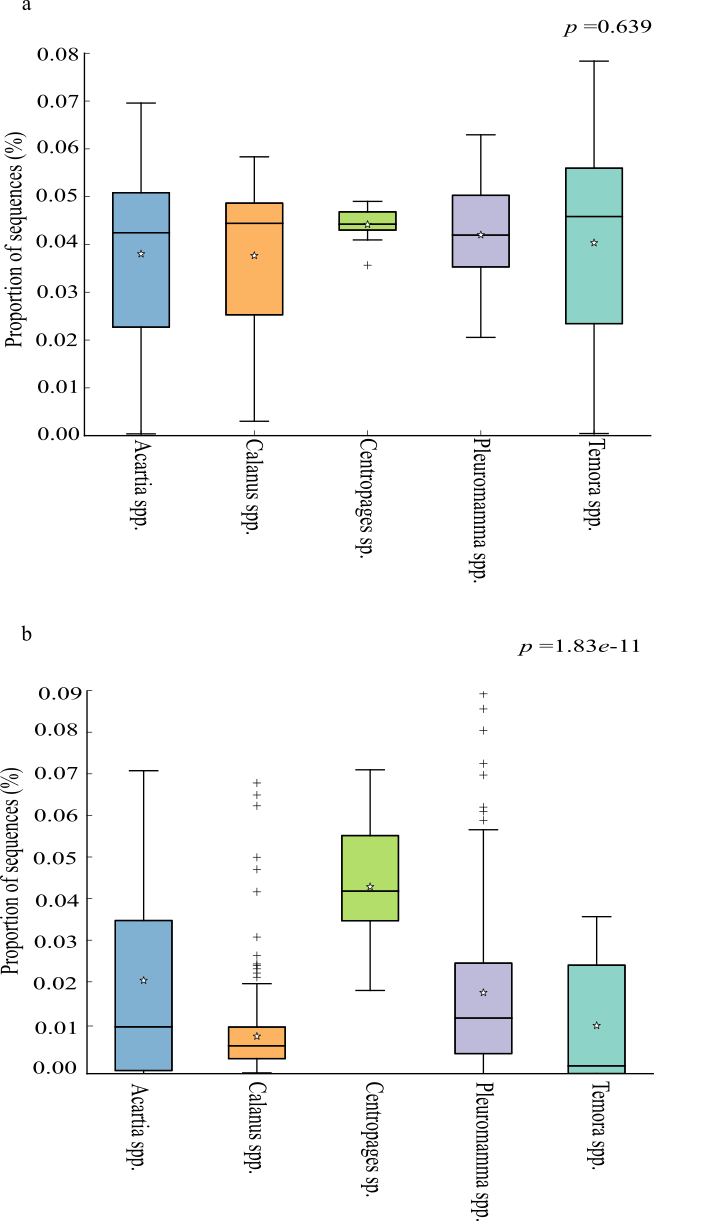


Figure S5. Relative abundances of a) PPC gene, b). Bacterial chitinase gene observed in CAB of copepods genera.


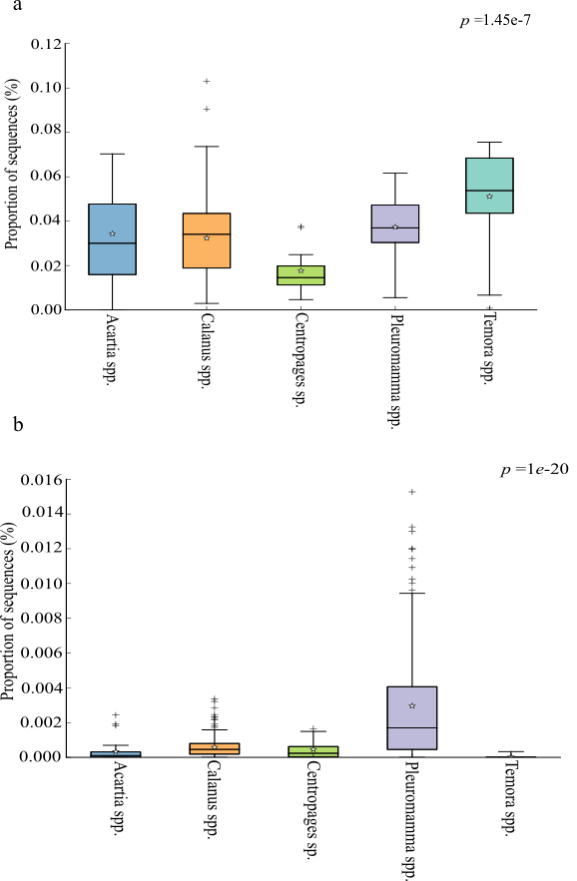


Figure S6. Relative proportions of a) feoA protein gene b) fhuF gene observed in CAB of copepods genera.


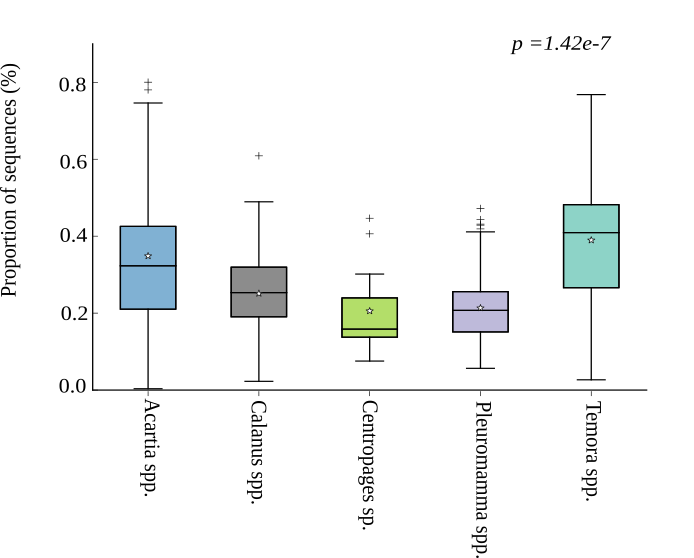


Figure S7. Relative proportions of potential Vitamin B12 synthesizing CAB of copepods genera.

| **W Valve** | **Reject null hypothesis** | **Centred log ratio (crl)** |
| --- | --- | --- |

| **Phylum** | **W Valve** | **Reject null hypothesis** | **Centred log ratio (crl)** |
| --- | --- | --- | --- |
| k__Bacteria;p__Cyanobacteria | 40 | True | 41.10 |
| k__Bacteria;p__Spirochaetes | 40 | True | 53.11 |
| k__Archaea;p__Crenarchaeota | 40 | True | 21.81 |
| k__Bacteria;p__Firmicutes | 40 | True | 13.11 |
| k__Bacteria;p__GN02 | 40 | True | 27.04 |
| k__Bacteria;p__Bacteroidetes | 40 | True | 17.94 |
| k__Bacteria;p__Proteobacteria | 40 | True | 13.18 |
| k__Bacteria;p__Planctomycetes | 39 | True | 4.72 |
| k__Bacteria;p__Actinobacteria | 39 | True | 17.07 |
| k__Bacteria;p__Acidobacteria | 39 | True | 15.93 |
| k__Archaea;p__Euryarchaeota | 39 | True | 17.93 |
| k__Bacteria;p__Verrucomicrobia | 38 | True | 15.84 |
| k__Bacteria;p__WPS-2 | 38 | True | 30.11 |
| k__Archaea;p__[Parvarchaeota] | 38 | True | 10.34 |
| k__Bacteria;p__[Thermi] | 38 | True | 10.29 |
| k__Bacteria;p__TM6 | 37 | True | 16.26 |
| k__Bacteria;p__Elusimicrobia | 35 | True | 14.03 |
| k__Bacteria;p__Fusobacteria | 35 | True | 6.12 |
| k__Bacteria;p__Chlorobi | 34 | True | 11.01 |
| k__Bacteria;p__Gemmatimonadetes | 32 | True | 8.42 |
| k__Bacteria;p__SBR1093 | 32 | True | 6.11 |
| k__Bacteria;p__Chlamydiae | 31 | True | 4.05 |
| k__Bacteria;p__OD1 | 30 | True | 2.68 |
| k__Bacteria;p__[Caldithrix] | 29 | False | 1.93 |
| k__Bacteria;p__TM7 | 29 | False | 2.06 |
| k__Bacteria;p__BRC1 | 25 | False | 6.06 |
| k__Archaea;p__ | 25 | False | 10.01 |
| k__Bacteria;__ | 25 | False | 6.80 |
| k__Bacteria;p__Tenericutes | 25 | False | 10.82 |
| k__Bacteria;p__Aquificae | 25 | False | 10.90 |
| k__Bacteria;p__Armatimonadetes | 24 | False | 9.17 |
| k__Bacteria;p__ | 24 | False | 5.03 |
| k__Bacteria;p__Fibrobacteres | 24 | False | 4.75 |
| k__Bacteria;p__Chloroflexi | 24 | False | 8.04 |
| k__Bacteria;p__OC31 | 24 | False | 7.03 |
| k__Bacteria;p__NKB19 | 24 | False | 8.40 |
| k__Bacteria;p__Lentisphaerae | 24 | False | 6.52 |
| k__Bacteria;p__SAR406 | 23 | False | 4.25 |
| k__Archaea;__ | 23 | False | 8.93 |
| k__Bacteria;p__SR1 | 18 | False | 0.72 |
| k__Bacteria;p__Nitrospirae | 18 | False | 0.41 |

**Table S1.** Differential percentile abundance of phyla observed in ANOCM analysis with W Statistical valve, Centred log ratio (crl) and hypothesis conditions.

Supplementary Excel files

1. Supplementary files Table S2. <https://figshare.com/articles/dataset/Supplementary_File-S1_xls/12612182>

2. Supplementary files Table S3. <https://figshare.com/articles/dataset/Supplementary-File-S2_xls/12612188>

**Abbreviation for the list of genes used in the study.**

dmdA; dimethylsulfoniopropionate-dependent demethylase A,

mttB; trimethylamine---corrinoid protein Co-methyltransferase,

mtsA; methylthiol:coenzyme M methyltransferase,

mtsB; methylated-thiol--corrinoid protein, sir; sulfite reductase (ferredoxin),

dmd-tmd; dimethylamine/trimethylamine dehydrogenase,

dmoA; dimethyl-sulfide monooxygenase,

phnJ; alpha-D-ribose 1-methylphosphonate 5-phosphate C-P lyase,

phnI; alpha-D-ribose 1-methylphosphonate 5-triphosphate synthase subunit,

PhnI, phnL; alpha-D-ribose 1-methylphosphonate 5-triphosphate synthase subunit,

PhnL, phnG; alpha-D-ribose 1-methylphosphonate 5-triphosphate synthase subunit, PhnG, phnM; alpha-D-ribose 1-methylphosphonate 5-triphosphate diphosphatase,

phnH; alpha-D-ribose 1-methylphosphonate 5-triphosphate synthase subunit,

PhnH, dddL; dimethylpropiothetin dethiomethylase,

dmsA; anaerobic dimethyl sulfoxide reductase subunit A,

dmsB; anaerobic dimethyl sulfoxide reductase subunit B,

dmsB*; dimethyl sulfoxide reductase iron-sulfur subunit,

dmsC*; dimethyl sulfoxide reductase membrane subunit,

dmsC; anaerobic dimethyl sulfoxide reductase subunit C,

norZ nitric oxide reductase subunit B,

nrfA; nitrite reductase,

nirS; nitrite reductase (NO-forming) / hydroxylamine reductase,

nirK; nitrite reductase (NO-forming),

norB; nitric oxide reductase subunit B,

norC; nitric oxide reductase subunit C,

narG; nitrate reductase / nitrite oxidoreductase, alpha subunit,

narH; nitrate reductase / nitrite oxidoreductase, beta subunit,

narH; nitrate reductase / nitrite oxidoreductase, beta subunit,

napA; nitrate reductase,

napB; nitrate reductase,

cysH; phosphoadenosine phosphosulphate reductase,

cysJ; sulfite reductase (NADPH) flavoprotein alpha-component,

cysI; sulfite reductase (NADPH) hemoprotein beta-component,

cysC; adenylylsulphate kinase,

nifH; nitrogenase iron protein,

nifK; nitrogenase molybdenum-iron protein beta chain,

nifD; nitrogenase molybdenum-iron protein alpha chain,

cysA; sulphate/thiosulphate transport system ATP-binding protein,

sat; sulphate adenylyltransferase,

cysD; sulphate adenylyltransferase subunit 2,

mxaF; methanol dehydrogenase (cytochrome c) subunit 1,

mxaI; methanol dehydrogenase (cytochrome c) subunit 2,

mtaA; [methyl-Co(III) methanol-specific corrinoid protein]:coenzyme M methyltransferase,

mtaB; methanol---5-hydroxybenzimidazolylcobamide Co-methyltransferase,

mcrA; methyl-coenzyme M reductase alpha subunit,

mtbC; dimethylamine corrinoid protein,

mtaC; methanol corrinoid protein.
